# Different influence of cardiac hemodynamics on thromboembolic events in patients with paroxysmal and non-paroxysmal atrial fibrillation

**DOI:** 10.1371/journal.pone.0214743

**Published:** 2019-03-29

**Authors:** Yun Gi Kim, Jaemin Shim, Ki Yung Boo, Do Young Kim, Suk-Kyu Oh, Kwang-No Lee, Jong-Il Choi, Young-Hoon Kim

**Affiliations:** Arrhythmia Center, Korea University Medical Center Anam Hospital, Seoul, Republic of Korea; Ziekenhuisgroep Twente, NETHERLANDS

## Abstract

Blood stasis in left atrium (LA) or LA appendage (LAA) is thought to be the main cause of thrombus formation and systemic embolism in atrial fibrillation (AF) patients. Paroxysmal and non-paroxysmal AF differ significantly in various aspects. Impact of cardiac hemodynamics on systemic embolism might also differ between the 2 distinct AF entities. This study was performed to evaluate the influence of cardiac hemodynamics on systemic embolism in both paroxysmal and non-paroxysmal AF. Consecutive AF patients undergoing radiofrequency catheter ablation (RFCA) in Korea University Medical Center Anam Hospital between June 1998 and February 2018 were analyzed. Among 2,801 patients who underwent first-time RFCA, a total of 231 patients had either previous ischemic stroke, transient ischemic attack, or arterial embolism. In paroxysmal AF, LA diameter, LA volume (measured with magnetic resonance imaging), left ventricular (LV) ejection fraction, E/e’, LAA flow velocity, and prevalence of spontaneous echocontrast (SEC) and dense SEC were significantly different between patients with and without thromboembolic events. However, only E/e’ was different between patients with and without thromboembolic events in non-paroxysmal AF. The influence of LA diameter, LA volume, LV EF, LAA flow velocity, and dense SEC on thromboembolic events was significantly moderated by the type of AF. In conclusion, paroxysmal and non-paroxysmal AF might have a different mechanism responsible for thrombus formation and consequent embolic events. Relative contribution of hemodynamic parameters and other factors such as atrial myopathy to thromboembolic events in paroxysmal versus non-paroxysmal AF needs further evaluation.

## Introduction

Thromboembolic events are major complications of atrial fibrillation (AF) [1, 2]. Blood stasis in left atrium (LA) and LA appendage (LAA) due to rapid and disorganized contraction is considered to be a main mechanism of thrombus formation and consequent embolization [3, 4]. The observation that decreased flow velocity in LAA and resulting spontaneous echocontrast (SEC) are associated with increased risk of thrombus formation and clinical events, such as ischemic stroke, supports this rhythm theory [3–5]. Recent studies suggest that radiofrequency catheter ablation (RFCA) in AF patients, which can cure AF or at least decrease the burden, is associated with significant reduction in the cumulative incidence of ischemic stroke [6, 7]. However, there are emerging evidences indicating atrial myopathy is the culprit pathology for thromboembolic complications in AF patients [8–10]. In the TREND study, more than 75% of AF patients who experienced ischemic stroke during thorough rhythm monitoring by implanted pacemakers, had no AF in the preceding 30 days [9]. The ASSERT trial also reported that 51% of patients who had ischemic stroke but without clinical AF experienced device-detected AF later during the follow up period and only 8% of patients with ischemic stroke had device-detected AF in the 30 days before the stroke [10]. Therefore, rhythm status cannot explain the underlying cause of thromboembolic complications in certain circumstances and according to the substrate theory, atrial fibrosis also known as atrial myopathy is the main cause of thrombus formation and subsequent adverse clinical events.

Paroxysmal and non-paroxysmal AF differs in various clinical and cardiac hemodynamic aspects. Notably, atrial fibrosis is the hallmark of non-paroxysmal AF and previous studies indicate increased burden of atrial fibrosis is clearly associated with increased risk of ischemic stroke [11, 12]. Therefore, the underlying mechanism of thromboembolic events in paroxysmal and non-paroxysmal AF might be different. We performed this analysis to compare the influence of various cardiac hemodynamic parameters on thromboembolic events in paroxysmal versus non-paroxysmal AF.

## Materials and methods

### Patients

RFCA registry of Korea University Medical Center Anam Hospital were utilized [13]. Patients who underwent first-time RFCA for AF between June 1998 and February 2018 in our institution were included and there was no specific exclusion criteria. Institutional Review Board of Korea University Medical Center Anam Hospital specifically approved this study. Written informed consent was waived because the current study was a retrospective analysis. The protocol of the current study was consistent with the ethical guidelines of the 2008 Helsinki Declaration. The aim of this study was to examine the underlying mechanism of thrombus formation and subsequent thromboembolic events in the two distinct type of AF: paroxysmal versus non-paroxysmal AF.

### Imaging evaluation

Transthoracic echocardiography (TTE) and transesophageal echocardiography (TEE) were performed prior to RFCA procedure and various echocardiographic parameters were measured. Diameter of LA, left ventricular (LV) ejection fraction, and E/e’ were measured by TTE. Presence of spontaneous echocontrast (SEC) or thrombus in LA or LAA was thoroughly examined. Emptying, filling, and average flow velocity of LAA were measured during TEE evaluation. In the main analysis, average flow velocity was used. In patients who performed cardiac magnetic resonance imaging (MRI) studies, LA volume was measured.

### Definitions

The current study evaluated the impact of various cardiac hemodynamic parameters measured with either echocardiography or cardiac MRI on previous thromboembolic events. Previous thromboembolic events were defined as confirmed prior diagnosis of ischemic stroke, transient ischemic attack, or arterial embolism. Ischemic stroke was defined as any neurologic symptoms lasting for more than 24 hours which cannot be explained by other medical conditions. Transient ischemic attack was defined as any neurologic symptoms which are not attributable to any other medical conditions which resolved completely within 24 hours. Arterial embolism was defined as occlusion of any artery that needed medical or surgical treatment. Modified CHA_2_DS_2_-VASc score (mCHA_2_DS_2_-VASc) was calculated as same method with CHA_2_DS_2_-VASc score except for excluding previous thromboembolic events for score summation. Paroxysmal AF was defined as AF episodes not lasting for more than 7 days. Non-paroxysmal AF was defined as AF episodes lasting for more than 7 days or requiring direct current cardioversion to terminate.

### Statistical analysis

Continuous variables are expressed as mean ± standard deviation (SD). Categorical variables are presented as percentile value. Unpaired t-test was used to compare continuous variables. Categorical variables were compared with either chi-square test or Fisher’s exact test as appropriate. Receiver operating characteristic (ROC) curve analysis was performed to calculate area under curve (AUC). Binary logistic regression analysis was performed to calculate odds ratio (OR) of thromboembolic events for each risk factor. OR was calculated separately for paroxysmal and non-paroxysmal AF and interaction term analysis was performed to evaluate whether the association between each risk factor and thromboembolic events was moderated by AF type. All significance tests were two-tailed and p values of less than 0.05 were considered statistically significant. All statistical analyses were performed with SPSS version 24.0 (SPSS Inc., Armonk, NY, USA).

### Data availability statement

All relevant data are within the paper and its Supporting Information files. The authors confirm that these data constitute the minimal data set required to support all the conclusions of the present study. However, the underlying individual-level patient data are restricted because they contain sensitive information such as age, sex, body weight, and medical comorbidities. The authors of the present study confirm that the de-identified individual patient data are not required to validate or confirm our study’s conclusions. However, these de-identified patient-level data will nevertheless be made available to interested researchers who meet the criteria for access to confidential information. These data can be requested from the Institutional Review Board of Korea University Medical Center Anam Hospital (+82-02-920-6566 or eirbadmin@kumc.or.kr).

## Results

### Patient characteristics

A total of 2,801 patients undergoing first-time RFCA were analyzed. Baseline characteristics of the study population are summarized in Table 1. Mean age was 55.58 ± 10.97 and 79.2% were male. Mean LA diameter was 41.16 ± 6.06 mm and 40.9% of patients had non-paroxysmal AF. CHA_2_DS_2_-VASc and mCHA_2_DS_2_-VASc scores were 1.25 ± 1.26 and 1.08 ± 1.10 respectively. Before undergoing RFCA, TTE, TEE, and cardiac MRI was performed in 2,742 (97.9%), 2,580 (92.1%), and 932 (33.3%) patients, respectively. A total of 1,064 patients underwent follow-up TTE (38.0%).

**Table 1:** Baseline clinical and echocardiographic characteristics.

	Total patients (N = 2,801)	Thromboembolic events (-) (n = 2,570)	Thromboembolic events (+) (n = 231)	p value
Clinical findings				
Age	55.58 ± 10.97	55.21 ± 11.10	59.68 ± 8.46	< 0.001
Male sex	2,217 (79.2%)	2,038 (79.3%)	179 (77.5%)	0.516
Body weight (kg)	70.80 ± 11.21	71.00 ± 11.29	68.62 ± 10.06	0.002
Height (cm)	168.18 ± 8.26	168.30 ± 8.29	166.85 ± 7.82	0.011
Body mass index (kg/m^2^)	24.97 ± 3.07	25.00 ± 3.08	24.61 ± 2.90	0.065
Heart failure	139 (5.0%)	124 (4.8%)	15 (6.5%)	0.263
Hypertension	1054 (37.6%)	957 (37.2%)	97 (42.0%)	0.153
Diabetes mellitus	266 (9.5%)	240 (9.3%)	26 (11.3%)	0.341
Vascular disease	226 (8.1%)	194 (7.5%)	32 (13.9%)	0.001
CHA_2_DS_2_-VASc	1.25 ± 1.26	1.06 ± 1.09	3.33 ± 1.14	< 0.001
mCHA_2_DS_2_-VASc	1.08 ± 1.10	1.06 ± 1.09	1.33 ± 1.14	< 0.001
Non-paroxysmal AF	1,145 (40.9%)	1,022 (39.8%)	123 (53.2%)	< 0.001
AF duration (years)	4.72 ± 4.62	4.70 ± 4.50	4.94 ± 5.77	0.524
Echocardiographic findings				
LA diameter (mm)	41.16 ± 6.06	41.06 ± 6.11	42.28 ± 5.37	0.001
LV ejection fraction (%)	54.77 ± 6.11	54.86 ± 6.05	53.80 ± 6.64	0.020
E/e’	8.79 ± 3.38	8.68 ± 3.27	10.02 ± 4.17	< 0.001
Pulmonary artery pressure (mmHg)	30.63 ± 5.25	30.62 ± 5.17	30.78 ± 6.01	0.684
LAA emptying velocity (cm/sec)	47.81 ± 21.81	48.28 ± 21.94	42.74 ± 19.73	< 0.001
LAA filling velocity (cm/sec)	49.28 ± 22.89	49.77 ± 23.00	43.95 ± 20.92	< 0.001
LAA average velocity (cm/sec)	48.55 ± 21.24	49.03 ± 21.36	43.34 ± 19.28	< 0.001
SEC	530 (20.6%)	471 (20.0%)	59 (27.1%)	0.013
Dense SEC	88 (3.4%)	75 (3.2%)	13 (6.0%)	0.031

AF: atrial fibrillation; LA: left atrium; LAA: left atrial appendage; LV: left ventricle; SEC: spontaneous echocontrast.

### History of previous thromboembolic events

Among 2,801 patients, 231 (8.3%) patients had a history of thromboembolic events. Clinical and echocardiographic characteristics of patients with and without previous thromboembolic events are summarized in Table 1. Patients with thromboembolic events showed significantly older age (55.21 ± 11.10 vs. 59.68 ± 8.46, p < 0.001), lower body weight (71.00 ± 11.29 vs. 68.62 ± 10.06, p = 0.002), small height (168.30 ± 8.29 vs. 166.85 ± 7.82, p = 0.011), and higher mCHA_2_DS_2_-VASc score (1.06 ± 1.09 vs. 1.33 ± 1.14, p < 0.001). Worse cardiac hemodynamics were observed in patients who experienced thromboembolic events: large LA diameter (41.06 ± 6.11 vs. 42.28 ± 5.37; p = 0.001), low LV ejection fraction (54.86 ± 6.05 vs. 53.80 ± 6.64, p = 0.020), high E/e’ (8.68 ± 3.27 vs. 10.02 ± 4.17; p < 0.001), low LAA flow velocity (49.03 ± 21.36 vs. 43.34 ± 19.28; p < 0.001), and higher prevalence of SEC (20.0% vs. 27.1%; p = 0.013) and dense SEC (3.2% vs. 6.0%; p = 0.031) (Table 1).

### Type of AF

Baseline characteristics of patients with paroxysmal AF and non-paroxysmal AF are presented in Table 2. Patients with non-paroxysmal AF showed significantly higher rate of previous thromboembolic events (6.5% vs. 10.7%; p < 0.001) despite similar mCHA_2_DS_2_-VASc score. Clinical heart failure was more prevalent in non-paroxysmal AF (3.1% vs. 7.6%; p < 0.001). The CHA_2_DS_2_-VASc score was higher and AF duration was longer in patients with non-paroxysmal AF. Cardiac hemodynamics including LA diameter, LA volume, LV ejection fraction, LAA flow velocity, SEC, and dense SEC were worse in patients with non-paroxysmal AF.

**Table 2:** Baseline characteristics between paroxysmal and non-paroxysmal AF.

	Paroxysmal AF (n = 1,656)	Non-paroxysmal AF (n = 1,145)	p value
Clinical findings			
Age	55.04 ± 11.42	56.37 ± 10.26	0.001
Male sex	1,269 (76.6%)	948 (82.8%)	< 0.001
Body weight (kg)	69.68 ± 11.07	72.40 ± 11.23	< 0.001
Height (cm)	167.86 ± 8.38	168.62 ± 8.06	0.018
Body mass index (kg/m^2^)	24.66 ± 2.98	25.41 ± 3.13	< 0.001
Heart failure	52 (3.1%)	87 (7.6%)	< 0.001
Hypertension	623 (37.6%)	431 (37.6%)	> 0.999
Diabetes mellitus	157 (9.5%)	109 (9.5%)	> 0.999
Thromboembolic events	108 (6.5%)	123 (10.7%)	< 0.001
Vascular disease	127 (7.7%)	99 (8.6%)	0.351
CHA_2_DS_2_-VASc	1.21 ± 1.21	1.31 ± 1.33	0.045
mCHA_2_DS_2_-VASc	1.08 ± 1.08	1.09 ± 1.12	0.733
AF duration (years)	4.30 ± 4.42	5.32 ± 4.83	< 0.001
Echocardiographic findings			
LA diameter (mm)	39.00 ± 5.26	44.20 ± 5.79	< 0.001
LV ejection fraction (%)	56.05 ± 4.99	52.97 ± 7.02	< 0.001
E/e’	8.68 ± 3.21	8.95 ± 3.60	0.051
Pulmonary artery pressure (mmHg)	30.71 ± 5.52	30.53 ± 4.89	0.423
LAA emptying velocity (cm/sec)	56.57 ± 20.50	36.45 ± 17.86	< 0.001
LAA filling velocity (cm/sec)	58.00 ± 21.67	37.97 ± 19.18	< 0.001
LAA average velocity (cm/sec)	57.28 ± 19.57	37.21 ± 17.68	< 0.001
SEC	138 (9.5%)	392 (35.0%)	< 0.001
Dense SEC	16 (1.1%)	72 (6.5%)	< 0.001
MRI findings			
LA volume (ml)	81.73 ± 26.29	107.96 ± 37.21	< 0.001
VENC (ml/sec)	61.95 ± 33.01	37.14 ± 27.98	< 0.001

AF: atrial fibrillation; LA: left atrium; LAA: left atrial appendage; LGE: late gadolinium enhancement; LV: left ventricle; MRI: magnetic resonance imaging; SEC: spontaneous echocontrast; VENC: velocity encoded cardiac MRI.

**Fig 1:**
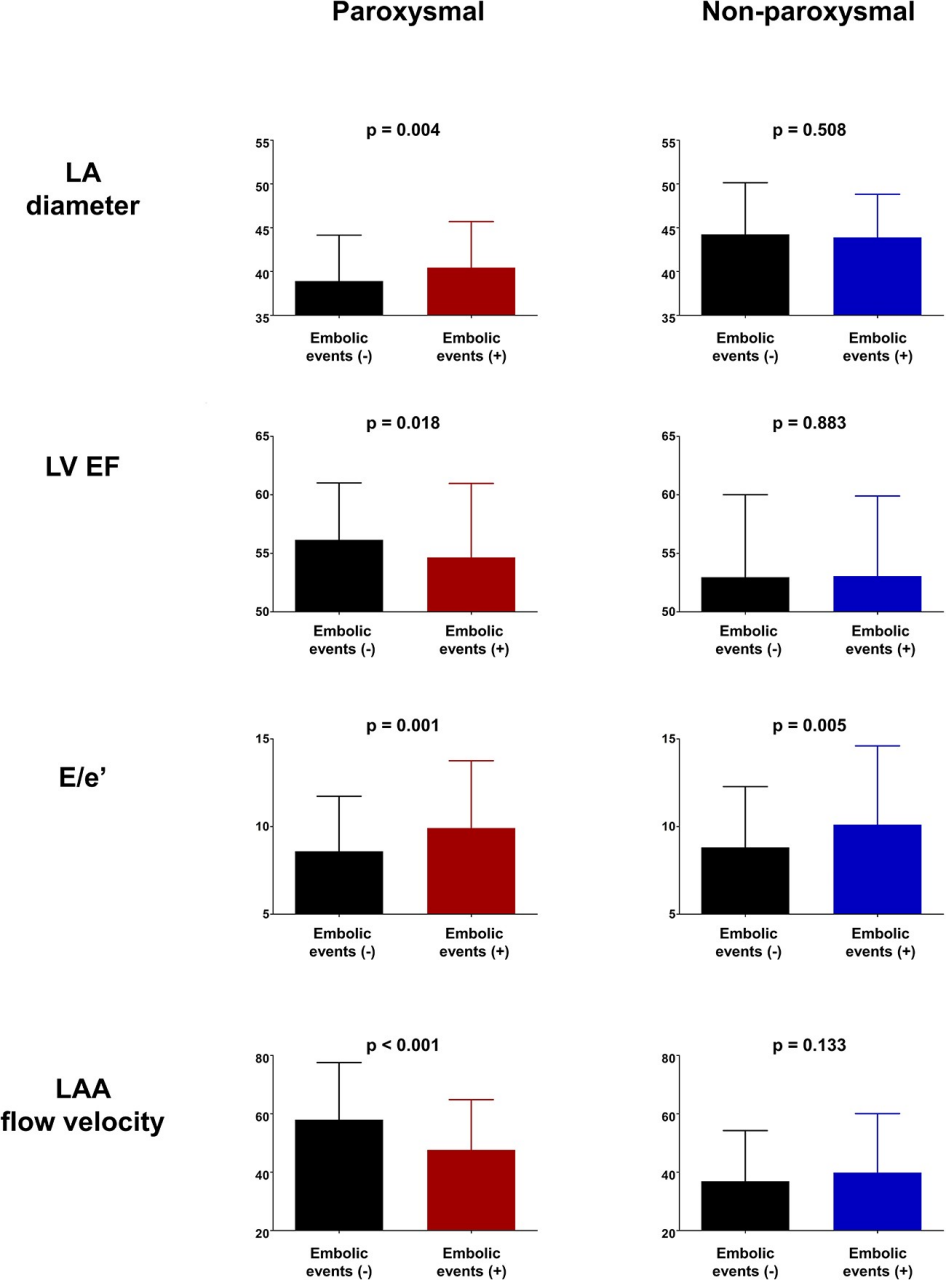
Thromboembolic events, various hemodynamic parameters, and AF type. In patients with paroxysmal AF, those with previous thromboembolic events had large LA, low LV EF, high E/e’, and decreased LAA flow velocity. However, no difference in LA diameter, LV EF, and LAA flow velocity was observed between between patients with and without history of thromboembolic events in non-paroxysmal type of AF. AF: atrial fibrillation; LA: left atrium; LAA: left atrial appendage; LV EF: left ventricular ejection fraction.

**Fig 2:**
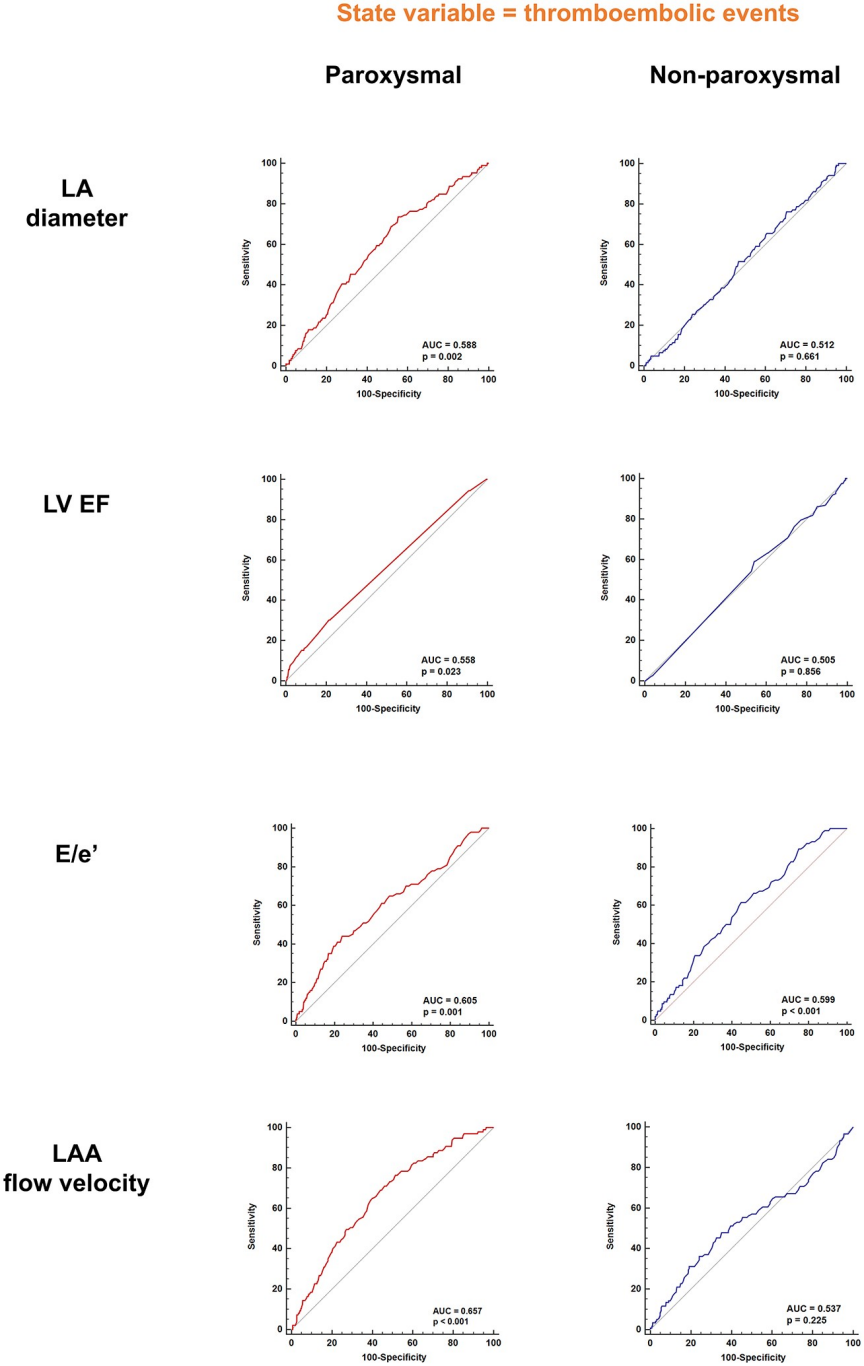
ROC curve analysis for cardiac hemodynamic parameters. LA diameter, LV EF, and LAA flow velocity was able to predict previous thromboembolic events only in paroxysmal type of AF. However, E/e’ was able to predict thromboembolic events in both paroxysmal and non-paroxysmal AF. AF: atrial fibrillation; LA: left atrium; LAA: left atrial appendage; LV EF: left ventricular ejection fraction; ROC: receiver operating characteristic.

In paroxysmal AF, patients who had previous thromboembolic events showed significantly large LA diameter (38.90 ± 5.25 vs. 40.44 ± 5.27, p = 0.004; Fig 1), lower LV ejection fraction (56.15 ± 4.87 vs. 54.65 ± 6.32, p = 0.018; Fig 1), higher E/e’ (8.59 ± 3.14 vs. 9.91 ± 3.83, p = 0.001; Fig 1), and lower LAA flow velocity (57.98 ± 19.55 vs. 47.67 ± 17.17, p < 0.001; Fig 1). In patients with non-paroxysmal AF, however, there were no significant differences in LA diameter (44.24 ± 5.89 vs. 43.88 ± 4.94, p = 0.508; Fig 1), LV ejection fraction (52.86 ± 7.05 vs. 53.06 ± 6.84, p = 0.883; Fig 1), and LAA flow velocity (36.89 ± 17.34 vs. 39.82 ± 20.23, p = 0.133; Fig 1) between patients with and without previous history of thromboembolic events. However, E/e’ was significantly higher in patients with previous thromboembolic events (8.81 ± 3.46 vs. 10.11 ± 4.49, p = 0.005; Fig 1). In ROC curve analysis, LA diameter, LV ejection fraction, and LAA flow velocity had statistically significant AUC only in paroxysmal AF patients (Fig 2). However, E/e’ showed significant predictive value for previous thromboembolic events in both paroxysmal and non-paroxysmal AF (Fig 2). The volume of LA measured with cardiac MRI also showed similar pattern as compared with LA diameter measured with TTE. The volume of LA had prognostic value for previous thromboembolic events only in paroxysmal AF (S1 and S2 Figs). In paroxysmal AF, patients with history of thromboembolic events showed significantly higher prevalence of both SEC (9.0% vs. 15.3%, p = 0.041; Table 3) and dense SEC (0.9% vs. 4.1%, p = 0.019; Table 3). In non-paroxysmal AF, however, no significant difference was observed in the prevalence of SEC (34.8% vs. 36.7%, p = 0.691; Table 3) and dense SEC (6.3% vs. 7.5%, p = 0.623; Table 3) between patients with and without thromboembolic events. Logistic regression analysis also revealed that SEC and dense SEC had increased odds ratio for previous thromboembolic events only in paroxysmal AF patients (Table 3). Odds ratios of each risk factor for thromboembolic events for both paroxysmal and non-paroxysmal AF patients are presented in Table 4. Type of AF demonstrated a significant moderator effect on individual risk factors for thromboembolic events (Table 4). However, the impact of E/e’ on thromboembolic events was not influenced by the type of AF and high E/e’ was a significant risk factor for thromboembolic events in both paroxysmal and non-paroxysmal AF patients. Change in LA diameter and LV ejection fraction before and after RFCA is summarized in S3 Fig.

**Table 3:** Relationship among SEC, type of AF, and thromboembolic events.

	Paroxysmal			Non-paroxysmal		
	**Thromboembolic events (-)**	**Thromboembolic events (+)**	**p value**	**Thromboembolic events (-)**	**Thromboembolic events (+)**	**p value**
**SEC**	123 (9.0%)	15 (15.3%)	0.041	348 (34.8%)	44 (36.7%)	0.691
**Dense SEC**	12 (0.9%)	4 (4.1%)	0.019	63 (6.3%)	9 (7.5%)	0.623
	**OR**	**95% CI**	**p value**	**OR**	**95% CI**	**p value**
**SEC**	1.818	1.017–3.247	0.044	1.083	0.731–1.605	0.691
**Dense SEC**	4.773	1.510–15.085	0.008	1.199	0.581–2.478	0.623

AF: atrial fibrillation; CI: confidence interval; OR: odds ratio; SEC: spontaneous echocontrast.

**Table 4:** Influence of AF type on the effect of each risk factors for thromboembolic events.

	Paroxysmal			Non-paroxysmal			Interaction	
	OR	95% CI	p value	OR	95% CI	p value	OR	p value
**LA diameter**	1.056	1.018–1.096	0.004	0.989	0.957–1.022	0.507	0.936	0.009
**LA volume**	1.012	1.002–1.022	0.016	0.996	0.987–1.006	0.440	0.985	0.024
**LAA flow velocity**	0.972	0.961–0.983	< 0.001	1.009	0.999–1.020	0.089	1.039	< 0.001
**E/e’**	1.105	1.050–1.162	< 0.001	1.082	1.034–1.133	0.001	0.979	0.549
**LV EF**	0.954	0.924–0.984	0.003	1.002	0.975–1.029	0.883	1.051	0.019
**Dense SEC**	4.773	1.510–15.085	0.008	1.199	0.581–2.478	0.623	0.251	0.047

AF: atrial fibrillation; CI: confidence interval; LA: left atrium; LAA: left atrial appendage, LV EF: left ventricular ejection fraction; OR: odds ratio; SEC: spontaneous echocontrast.

## Discussion

The main findings of the current study can be summarized as follows: (i) increased LA diameter, decreased LV ejection fraction, decreased LAA flow velocity, and presence of SEC or dense SEC are risk factors for thromboembolic events only in patients with paroxysmal AF; (ii) increased E/e’ is a risk factor for thromboembolic events regardless of AF type; (ii) impact of aforementioned risk factors on thromboembolic events, except for E/e’, is significantly moderated by the type of AF. This is the first study to analyze the impact of multiple TTE and TEE parameters, which reflects various aspects of cardiac hemodynamics, on thromboembolic risk in patients with paroxysmal and non-paroxysmal AF.

### Paroxysmal vs. non-paroxysmal

The major difference between paroxysmal AF and non-paroxysmal AF is whether AF is sustained [14–16]. In order to sustain AF, atrial remodeling which is often called as ‘substrate’ is required [14–16]. Therefore, non-paroxysmal AF, by its definition, has more substrate than paroxysmal AF which is demonstrated by significant lower LA voltage in non-paroxysmal AF even after LA volume adjustment [17]. In cardiac MRI evaluation, increased late gadolinium enhancement which is a reliable marker for atrial fibrosis, is observed with higher degree in patients with non-paroxysmal AF as compared with paroxysmal AF [11]. Increased amount of atrial fibrosis is also shown to be associated with increased risk of thromboembolic events [11]. In the current study, patients with non-paroxysmal AF had significantly higher prevalence of previous thromboembolic events. Our study also revealed that non-paroxysmal AF has worse cardiac hemodynamics such as enlarged LA, low LV ejection fraction, decreased LAA flow velocity, and higher prevalence of SEC and dense SEC as compared with paroxysmal AF. These parameters, however, was associated with increased risk of thromboembolic events only in paroxysmal AF and not in non-paroxysmal AF suggesting that another underlying mechanism for thrombus formation and embolization is present for non-paroxysmal AF patients.

### Influence of AF type

In the current analysis, most of echocardiographic risk factors for thromboembolic events, such as LA size, LV ejection fraction, LAA flow velocity, or SEC, were only valid in paroxysmal AF and impact of these risk factors on thromboembolic events was clearly moderated by the type of AF. In contrast, increased E/e’ was a clear risk factor for thromboembolic events regardless of AF type. Mechanism of thrombus formation in LA or LAA is usually explained by 2 theories: rhythm or substrate. In the rhythm theory, rapid and disorganized contraction of LA and LAA result in blood stasis which in turn, provide a nice nidus for SEC and thrombus formation [3, 4]. The substrate theory, however, proposes that atrial fibrosis increases the risk of thromboembolic events independently of atrial rhythm status [8, 18, 19]. Increased atrial fibrosis might reduce contractility of LA and LAA which will in turn, results in increased LA size, decreased LAA blood flow, and formation of SEC. Furthermore, replacement of normal myocyte lining in the endocardium with fibrotic tissue might provoke coagulation cascade and initiate thrombosis formation irrespective of cardiac hemodynamics. Our data support this concept. In patients with non-paroxysmal AF, markers of cardiac hemodynamics, except for E/e’, were not useful to identify patients at higher risk of thromboembolic events. Therefore, it might be the atrial fibrosis itself rather than cardiac hemodynamics which is the main driver of thrombus formation and consequent thromboembolic events in patients with non-paroxysmal AF.

Atrial fibrosis observed in AF patients is characterized by excessive accumulation of collagenous material in the extracellular space [18, 20, 21]. In the endovascular system, collagen, which is highly thrombogenic, is exposed to blood after endothelial disruption, starting the formation of a thrombus [22]. Collagen material can have similar effect in cardiac chambers. Replacement of normal atrial myocyte with collagen material might increase thrombogenecity by not only decreasing LA function but also by thrombogenic effect of collagen itself. The impact of guiding anticoagulation treatment based on the degree of atrial fibrosis should be examined in future clinical trials.

In addition to rhythm and substrate, abnormality of blood constituents is another important factor associated with thromboembolic events in AF patients [23]. Elevation patterns of Von Willebrand factor, fibrinogen, and P-selectin differed among paroxysmal, persistent, and permanent AF according to the previous study [24]. Furthermore, the duration of AF was independently associated with abnormal measured factors [24]. Therefore, paroxysmal and non-paroxysmal AF might have different degree of abnormality of blood constituents which might explain different impact of hemodynamic parameters on thromboembolic events in paroxysmal versus non-paroxysmal AF in this study. Inflammatory markers such as C-reactive protein or interleukin-6 are also associated with prothrombotic state in patients with AF [23, 25]. Whether these inflammatory markers have different role in paroxysmal versus non-paroxysmal AF is an area of future research.

## Limitations

The current study has several limitations. First, TTE and TEE evaluations were done after the occurrence of previous thromboembolic events. Second, previous thromboembolic events were diagnosed based on patient history and medical records rather than imaging modalities. Third, although total patient number was quite large, the sample size of patients with previous history of thromboembolic events was of moderate number. Fourth, this study included only patients undergoing RFCA for AF and therefore, do not reflect the whole AF patient. Our study population was consisted of East Asian patients and caution is needed when applying our results to different ethnicity. Fifth, AUC of individual TTE and TEE risk factors was not high and therefore, the risk of thromboembolic events cannot be fully explained by individual hemodynamic parameters. It will be helpful to take into account multiple cardiac hemodynamic parameters comprehensively in addition to clinical factors and markers of atrial myopathy when estimating future risk of thromboembolic events in a given AF patient.

## Conclusions

Hemodynamic parameters of LA and LAA had different impact on thromboembolic events between patients with paroxysmal and non-paroxysmal AF. This study suggests that mechanisms of thrombus formation and subsequent thromboembolic events in the two distinct type of AF might be different.

## Supporting information

S1 Fig LA volume as a risk factor for thromboembolic events. In paroxysmal AF, patients with previous thromboembolic events showed significantly large LA volume which was measured with cardiac MRI. However, LA volume was not different between patients with and without thromboembolic events in non-paroxysmal AF. AF: atrial fibrillation; LA: left atrium; MRI: magnetic resonance imaging.
(TIF)

S2 Fig ROC curve analysis. LA volume was able to predict previous thromboembolic events only in paroxysmal AF patients. AF: atrial fibrillation; LA: left atrium; ROC: receiver operating characteristic.
(TIF)

S3 Fig Change in LA diameter and LV EF before and after RFCA. LA diameter was decreased after ablation. LV EF was increased after ablation but the degree of improvement was negligible. AF: atrial fibrillation; LA: left atrium; LV EF: left ventricular ejection fraction; RFCA: radiofrequency catheter ablation.
(TIF)

## Abbreviations

AF: atrial fibrillation
AUC: area under curve
CT: computed tomography
HR: hazard ratio
LA: left atrium
LAA: left atrial appendage
mCHA_2_DS_2_-VASc: modified CHA_2_DS_2_-VASc
MRI: magnetic resonance imaging
OR: odds ratio
RFCA: radiofrequency catheter ablation
ROC: receiver operating characteristic
SEC: spontaneous echocontrast
TEE: transesophageal echocardiography
TTE: transthoracic echocardiography
