# Supplementary figures and images for: Different influence of cardiac hemodynamics on thromboembolic events in patients with paroxysmal and non-paroxysmal atrial fibrillation
Source: PLoS One. 2019 Mar 29;14(3):e0214743. doi: 10.1371/journal.pone.0214743 (PMC6440630; doi:10.1371/journal.pone.0214743)

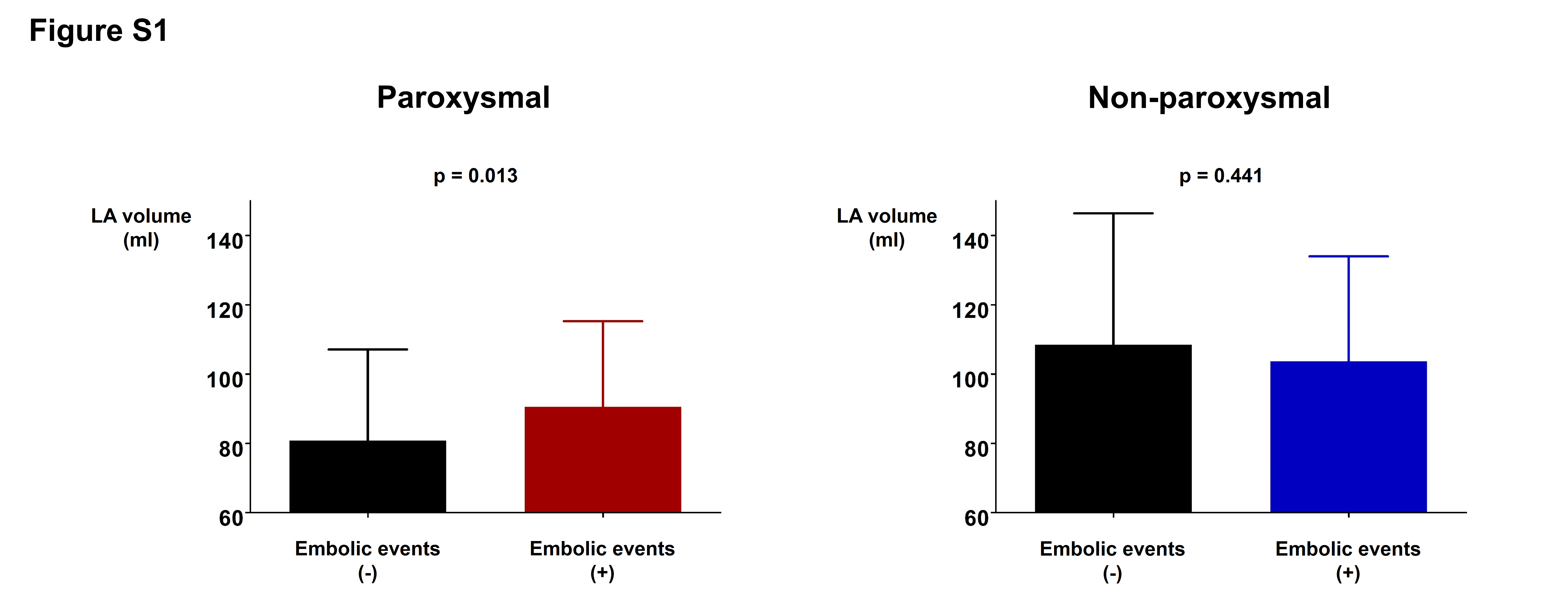

Supplement: S1 Fig — In paroxysmal AF, patients with previous thromboembolic events showed significantly large LA volume which was measured with cardiac MRI. However, LA volume was not different between patients with and without thromboembolic events in non-paroxysmal AF. AF: atrial fibrillation; LA: left atrium; MRI: magnetic resonance imaging. (TIF) [file pone.0214743.s001.tif]

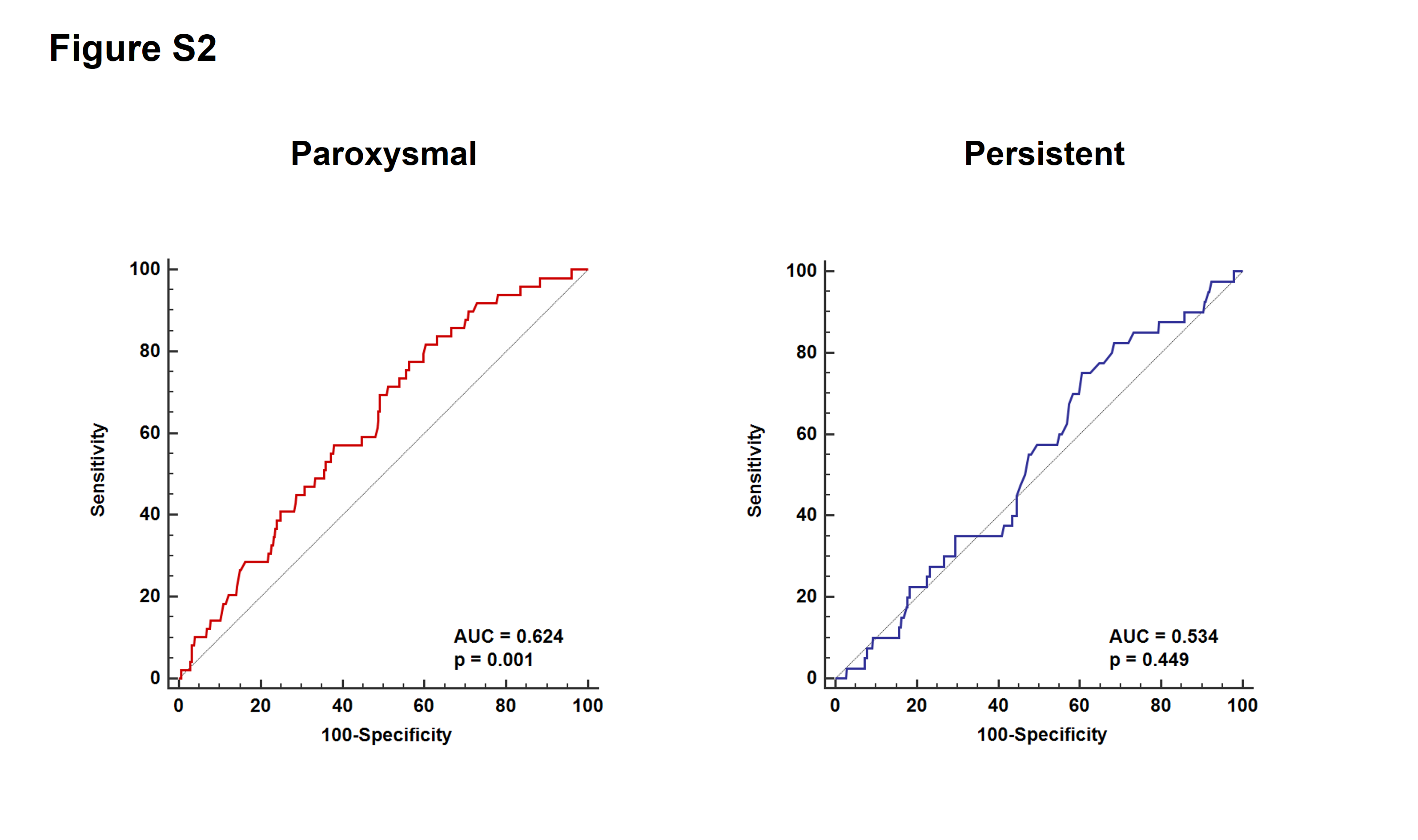

Supplement: S2 Fig — LA volume was able to predict previous thromboembolic events only in paroxysmal AF patients. AF: atrial fibrillation; LA: left atrium; ROC: receiver operating characteristic. (TIF) [file pone.0214743.s002.tif]

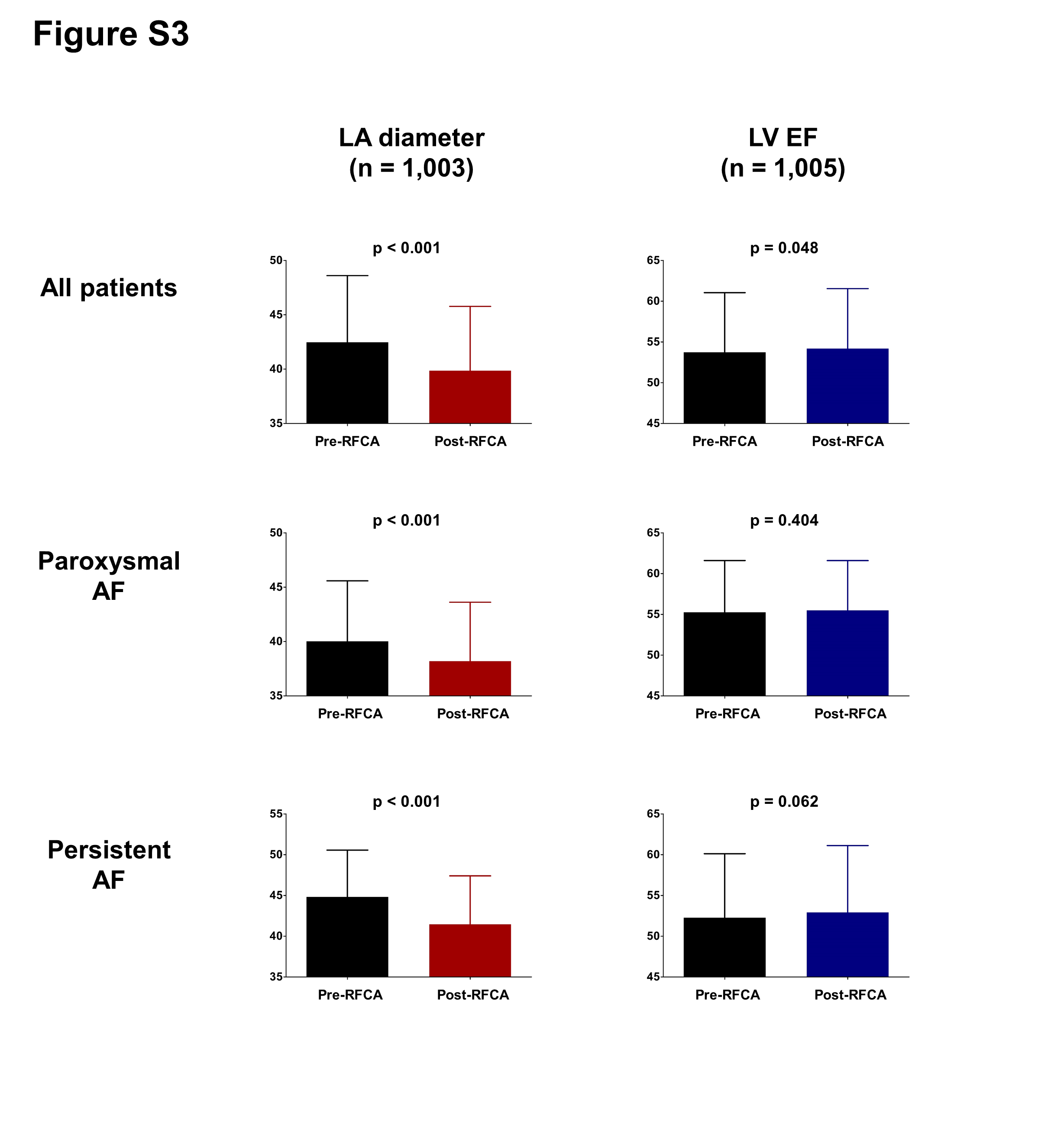

Supplement: S3 Fig — LA diameter was decreased after ablation. LV EF was increased after ablation but the degree of improvement was negligible. AF: atrial fibrillation; LA: left atrium; LV EF: left ventricular ejection fraction; RFCA: radiofrequency catheter ablation. (TIF) [file pone.0214743.s003.tif]
